# The Spl Serine Proteases Modulate *Staphylococcus aureus* Protein Production and Virulence in a Rabbit Model of Pneumonia

**DOI:** 10.1128/mSphere.00208-16

**Published:** 2016-10-12

**Authors:** Alexandra E. Paharik, Wilmara Salgado-Pabon, David K. Meyerholz, Mark J. White, Patrick M. Schlievert, Alexander R. Horswill

**Affiliations:** a Department of Microbiology, Carver College of Medicine, University of Iowa, Iowa City, Iowa, USA; b Department of Pathology, Carver College of Medicine, University of Iowa, Iowa City, Iowa, USA; University of Kentucky

**Keywords:** *Staphylococcus aureus*, mucin, pneumonia, proteases, Spl, virulence factors

## Abstract

The Spl proteases are a group of six serine proteases that are encoded on the νSaβ pathogenicity island and are unique to *Staphylococcus aureus*. Despite their interesting biochemistry, their biological substrates and functions in virulence have been difficult to elucidate. We found that an *spl* operon mutant of the community-associated methicillin-resistant *S. aureus* USA300 strain LAC induced localized lung damage in a rabbit model of pneumonia, characterized by bronchopneumonia observed histologically. Disease in the mutant-infected rabbits was restricted in distribution compared to that in wild-type USA300-infected rabbits. We also found that SplA is able to cleave the mucin 16 glycoprotein from the surface of the CalU-3 lung cell line, suggesting a possible mechanism for wild-type USA300 spreading pneumonia to both lungs. Investigation of the secreted and surface proteomes of wild-type USA300 and the *spl* mutant revealed multiple alterations in metabolic proteins and virulence factors. This study demonstrates that the Spls modulate *S. aureus* physiology and virulence, identifies a human target of SplA, and suggests potential *S. aureus* targets of the Spl proteases.

**IMPORTANCE** *Staphylococcus aureus* is a versatile human pathogen that produces an array of virulence factors, including several proteases. Of these, six proteases called the Spls are the least characterized. Previous evidence suggests that the Spls are expressed during human infection; however, their function is unknown. Our study shows that the Spls are required for *S. aureus* to cause disseminated lung damage during pneumonia. Further, we present the first example of a human protein cut by an Spl protease. Although the Spls were predicted not to cut staphylococcal proteins, we also show that an *spl* mutant has altered abundance of both secreted and surface-associated proteins. This work provides novel insight into the function of Spls during infection and their potential ability to degrade both staphylococcal and human proteins.

## Abstract (precis)

*Staphylococcus aureus* is a versatile human pathogen that produces an array of virulence factors, including several proteases. Of these, six proteases called the Spls are the least characterized. Previous evidence suggests that the Spls are expressed during human infection; however, their function is unknown. Our study shows that the Spls are required for *S. aureus* to cause disseminated lung damage during pneumonia. Further, we present the first example of a human protein cut by an Spl protease. Although the Spls were predicted not to cut staphylococcal proteins, we also show that an *spl* mutant has altered abundance of both secreted and surface-associated proteins. This work provides novel insight into the function of Spls during infection and their potential ability to degrade both staphylococcal and human proteins.

## INTRODUCTION

*Staphylococcus aureus* is a Gram-positive opportunistic pathogen that is a significant cause of both health care- and community-associated infectious disease and is responsible for nearly 500,000 hospitalizations per year in the United States (1). This versatile organism uses a wide array of virulence factors to cause many types of infections, including cutaneous lesions, pneumonia, osteomyelitis, and toxic shock syndrome (2). Although many virulence factors have been characterized, *S. aureus* produces other putative virulence factors that are poorly studied. One example is the *spl* (serine protease-like) operon, which is found on the νSaβ pathogenicity island and carries six serine protease genes (*splA*, *splB*, *splC*, *splD*, *splE*, and *splF*) (3, 4). The *spl* operon is not found in the other staphylococci but is present in most strains of *S. aureus*, although some strains do not have the full operon (3, 5). The SplD and SplF amino acid sequences are 94.6% identical (3). The natural substrates and virulence roles of the Spl proteases are unknown; however, there is evidence that they are involved in colonization or infection of the host.

The *spl* locus was first identified in a study of anti-*S. aureus* antibodies generated in patients with invasive *S. aureus* infections. In a screen of *S. aureus* proteins, one that had strong reactivity with the patients’ antisera was characterized. Sequence analysis of the open reading frame (ORF) identified revealed that it was a putative serine protease and appeared to be within an operon (6). The ORF identified was later found to be SplC (3). Since then, the Spls have been demonstrated to be immunogenic in individuals with *S. aureus* infections, as well as healthy individuals colonized by *S. aureus* (5, 7). A recent study also identified the Spls as immunogenic in the airway, particularly in patients with severe asthma (8). Strikingly, the same study also discovered peptide fragments of SplD and SplF in human nasal polyp tissue. Therefore, it is well established that Spl proteases are secreted *in vivo* and are potentially involved in *S. aureus*-host interaction.

The goal of this study was to investigate the role of the Spl proteases in infection and identify possible Spl cleavage targets. Using a community-associated methicillin-resistant *S. aureus* (CA-MRSA) USA300 strain, we found that an *spl* deletion mutant was not attenuated in lethality in a rabbit pneumonia model but was able to induce severe damage in only one lung. In contrast, the USA300 wild-type (WT) strain induced more diffuse disease, affecting both lungs equally. We also demonstrated that SplA is able to cleave mucin 16 from the human lung cell line CalU-3, which is the first identification of a host protein as a substrate for an Spl protease. Proteomic studies show that the *spl* mutant has an altered abundance of many proteins both on the cell surface and secreted, suggesting that Spls may target *S. aureus* proteins as well.

## RESULTS

### *spl* mutant produces localized pneumonia in rabbit model of infection.

To study the function of the Spls during host infection, we compared the USA300 WT strain LAC and an allelic replacement *spl* operon mutant (Δ*spl*::*erm*) in a rabbit pneumonia model of infection. LAC is a CA-MRSA USA300 strain (here USA300 WT), and this USA300 lineage has become the most frequent cause of CA-MRSA infections in North America and is increasingly reported both overseas and in hospital-associated MRSA (HA-MRSA) infections (9, 10). USA300 strains have been isolated from both CA-MRSA and HA-MRSA pneumonia patients (11, 12). Animal models have demonstrated that the *S. aureus sae* regulatory system and various *sae-*regulated factors are important in *S. aureus* pneumonia (13–17). Since the Spls are directly regulated by *sae* (13), we predicted that pneumonia would be a relevant model to investigate their role in virulence.

**FIG 1:**
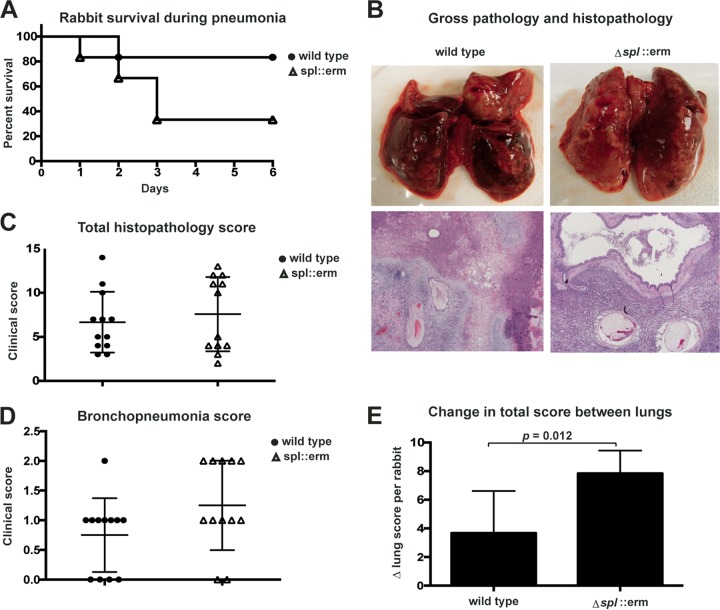
USA300 *spl* mutant causes unilateral pneumonia. (A) Survival of rabbits infected with the USA300 WT or *spl* mutant strain (*n* = 6). Two experiments were performed, with three rabbits per group in each experiment. The *P* value (0.11) was calculated with a log rank (Mantel-Cox) test. (B) Gross pathology (top) and histopathology (bottom) of USA300 WT and *spl* mutant strain-infected rabbit lungs euthanized on day 6. Histopathology of H&E-stained infected lung tissue is shown. (C) Total histopathology score of each lung, combined from six scoring categories. *n =* 12 lungs per group. (D) Bronchopneumonia score of each lung. *n =* 12 lungs per group. *P* = 0.09 calculated with an unpaired, two-tailed *t* test. (E) Difference between the left and right lung scores of each rabbit. For each rabbit, the value of the lung with the lower total clinical score was subtracted from the value of the lung with the higher clinical score. *n =* 6 rabbits per group. The *P* value was calculated with an unpaired, two-tailed *t* test.

The rabbit pneumonia model was performed as previously described (18). Rabbits were infected with either USA300 WT or Δ*spl*::*erm* mutant bacteria at a dose of 2 × 10^9^ CFU delivered directly to the lung through a catheter that was inserted at the trachea. After 6 days of infection, surviving rabbits were sacrificed. In a total of two experiments (*n =* 6 rabbits per group), 83% of the USA300 WT-infected rabbits (5 of 6) survived to day 6, while 33.3% of the *spl* mutant-infected rabbits (2 of 6) survived to day 6 (*P* = 0.11, Fig. 1A). Gross pathology and histopathology revealed significant lung damage in both groups. Gross pathology of a USA300 WT-infected rabbit that was euthanized on day 6 showed hemorrhage in both the left and right lungs (Fig. 1B, upper left). In a Δ*spl*::*erm* mutant-infected animal that also was euthanized on day 6, the left lung also showed a large area of hemorrhage, while the right lung appeared more intact, suggesting a more constrained distribution of lesions (Fig. 1B, upper right). Histopathology also revealed edema, inflammatory infiltrates, and pleuritis in infected lungs from both groups of animals. Hematoxylin and eosin (H&E) straining of the left lungs of USA300 WT-infected (Fig. 1B, lower left) and Δ*spl*::*erm* mutant-infected (Fig. 1B, lower right) rabbits euthanized on day 6 demonstrated these findings in both animals.

Histopathology scoring of the lungs of all of the infected rabbits in both groups was also performed, regardless of the time of death. The lungs were scored on the presence of pleuritis, edema, bronchopneumonia, necrosis, lymphoid cell/heterophil infiltration, and Gram-positive bacteria. Each of the six categories was given a score of 0 (within normal limits) to 3 (severe and extensive). The average total histopathology scores were nearly identical in WT strain-infected rabbits and Δ*spl*::*erm* mutant-infected rabbits (Fig. 1C), and rabbits in both groups exhibited bronchopneumonia (Fig. 1D). Interestingly, the Δ*spl*::*erm* mutant-infected rabbits displayed a pattern of one high-scoring lung and one low-scoring lung, while lung scores appeared more evenly distributed in the WT strain-infected rabbits (Fig. 1C and E). A D’Agostino-Pearson normality test determined that the WT strain-infected lung scores follow a normal distribution (*P* = 0.27), while mutant-infected lung scores do not (*P* = 0.01), suggesting that the mutant-infected lungs represent two distinct populations. When the difference in score between the two lungs was calculated (Δ lung score) for each rabbit, the *spl* mutant-infected rabbits had a significantly greater difference between their lungs than WT strain-infected rabbits did (Fig. 1E, *P* = 0.012). On the whole, these findings indicate that although no significant difference in the lethality of the Δ*spl*::*erm* mutant was observed, this mutant also produced a clear phenotype of lung damage that has a constrained localization within the lungs.

### SplA induces mucin 16 release from CalU-3 cells.

Mucin 16 is an ~25-MDa, heavily glycosylated cell surface protein that is found on multiple epithelial tissues in the body, including the ocular surface and airway epithelia (19–21). Like other mucins, mucin 16 provides lubrication of epithelia, as well as a protective barrier against pathogens. Two studies have reported that RNA interference-mediated knockdown of mucin 16 in human corneal-limbal epithelial cells renders these cells more susceptible to *S. aureus* adherence (22, 23). Additionally, the *Streptococcus pneumoniae* metalloprotease ZmpC has been shown to cleave mucin 16 from a variety of human epithelial cell types, allowing the bacteria to increasingly invade these cells *in vitro* (24). An adenovirus capable of causing keratoconjunctivitis also was recently shown to induce mucin 16 release from ocular epithelial cells, facilitating infection (25). These findings demonstrate that *S. aureus* and other pathogens encounter mucin 16 as a barrier to the host epithelium and are able to disrupt this barrier in order to colonize or infect the host.

**FIG 2:**
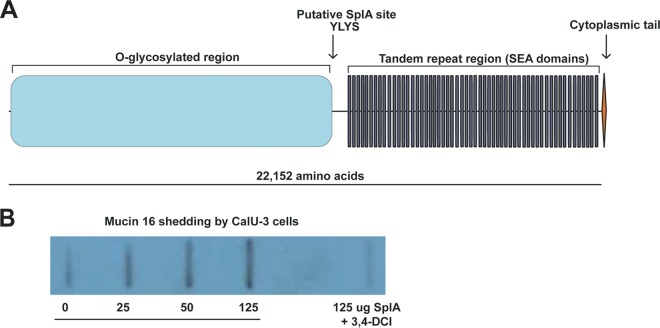
SplA cleaves human mucin 16. (A) Diagram of human mucin 16. Mucin 16 is exposed on the cell surface and contains one putative SplA cleavage site. The N-terminal portion is heavily glycosylated, and the tandem repeat region contains several SEA (sea urchin sperm protein, enterokinase, and agrin) domains, which are present in various mucins. (B) Anti-mucin 16 slot blot assay of CalU-3 medium following treatment with SplA. One representative blot is shown.

A study describing the crystal structure and cleavage site preference of SplA found that this enzyme has highly specific substrate requirements. In an experiment with a cellular library of peptide substrates, SplA was found to cleave exclusively substrates containing the Trp/Tyr-Leu-Tyr-Thr/Ser (W/Y-L-Y-T/S) motif (26). That study also noted that mucin 16 contains a Y-L-Y-S sequence and could be a likely target for SplA cleavage (Fig. 2A) (26). On the basis of this evidence, as well as our observation that the Δ*spl*::*erm* mutant exhibited more localized disease in the rabbit pneumonia model, we hypothesized that SplA cleaves mucin 16 from lung cells. To test this, confluent monolayers of the lung epithelial cell line CalU-3 were treated with purified, His6-tagged SplA. Because of the large size of mucin 16 (Fig. 2A), CalU-3 medium was tested for mucin 16 with a slot blot apparatus rather than by gel electrophoresis. Probing with a mucin 16 antibody revealed that mucin 16 is endogenously released from CalU-3 cells (Fig. 2B). This is in accordance with previous reports that mucin 16 is naturally shed from epithelia (19, 27, 28). Incubation with SplA increased this shedding in a dose-dependent manner. When SplA was treated with the serine protease inhibitor 3,4-dichloroisocoumarin (3,4-DCI) before it was added to the cells, mucin 16 shedding was decreased to a level similar to that in untreated cells (Fig. 2B). This suggests that SplA activity is required for mucin 16 removal, by either cleaving it at the Y-L-Y-S site or inducing its shedding indirectly by degradation of another substrate. This activity may allow *S. aureus* to perturb the airway epithelial barrier and cause more disseminated disease.

### The Δ*spl*::*erm* mutant retains viability in whole blood, hemolysis activity, and protease production.

**FIG 3:**
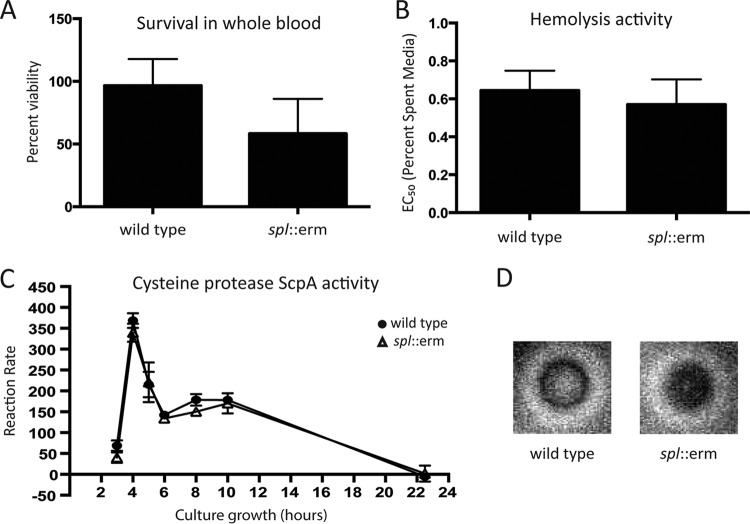
Virulence factor production is unchanged in a USA300 *spl* mutant. (A) Survival of the USA300 WT and Δ*spl*::*erm* mutant strains in whole human blood. Bacteria were incubated with whole blood for 3 h. (B) Hemolysis activities of the USA300 WT and Δ*spl*::*erm* mutant strains. A dilution series of spent medium was incubated with rabbit erythrocytes. The graph shows the percentage of medium needed to lyse 50% of the erythrocytes (EC_50_). (C) ScpA activity of the USA300 WT and Δ*spl*::*erm* mutant strains measured over the course of culture growth. At each time point, spent medium was incubated with a FRET substrate for ScpA activity. Activity is shown as the rate at which fluorescence was generated by FRET substrate cleavage. (D) Milk plate proteolysis activities of the USA300 WT and Δ*spl*::*erm* mutant strains*.* Overnight cultures were spotted onto an agar plate containing 5% milk, and the halo around the colony corresponds to clearing of the milk.

*S. aureus* produces a multitude of virulence factors that function by thwarting the innate immune defenses of the infected host (29). Based on the *in vivo* phenotype of the Δ*spl*::*erm* mutant, we hypothesized that it may have an altered capacity to survive these immune assaults. To test this, we compared the survival of USA300 WT and Δ*spl*::*erm* mutant cells in whole human blood. Whole human blood contains cellular and complement-mediated innate immunity components and thus is used as a general assay of bacterial survival under these conditions. When log-phase bacteria were inoculated into whole human blood and incubated for 3 h, 60% of USA300 WT bacteria remained viable. However, 40% of Δ*spl*::*erm* mutant bacteria remained viable (Fig. 3A), although this difference was not statistically significant.

Alternatively, we hypothesized that the Δ*spl*::*erm* mutant had altered production of one or more secreted virulence factors whose levels could be modulated by proteolytic degradation by the Spl proteases in *S. aureus*. To investigate this, the activity of the secreted alpha toxin (Hla) and the secreted proteases staphopain A (ScpA) and aureolysin (Aur) were compared in USA300 WT and Δ*spl*::*erm* mutant bacteria. Hla is a potent cytolysin and immunomodulatory virulence factor that has been shown to contribute to pathogenesis in a number of *S. aureus* infection models, including a murine model of pneumonia (30, 31). In an Hla activity assay (32), the Δ*spl*::*erm* mutant did not exhibit a change in Hla activity from the USA300 WT strain (Fig. 3B), indicating that hemolysis is unaffected by the Spls.

Next, we tested the activity levels of the *S. aureus* secreted proteases staphopain A (ScpA) and aureolysin (Aur). Both of these proteases inhibit the classical and alternative pathways of complement activation (33). ScpA also cleaves the neutrophil chemotaxis receptor CXCR2, blocking neutrophil recruitment (34), and exhibits activity against the extracellular matrix protein collagen, whose degradation could promote host damage during infection (35). Aur is able to inhibit complement activation by cleaving the complement protein C3 (36). ScpA activity in cell-free spent medium was tested with a FRET substrate containing the ScpA cleavage site from human CXCR2 (34, 37). Over the course of growth, the Δ*spl*::*erm* mutant displayed the same ScpA activity as the USA300 WT strain, indicating that the Spls do not affect ScpA activity (Fig. 3C). A milk plate assay to detect Aur activity showed no decrease in the Δ*spl*::*erm* mutant (Fig. 3D). On the whole, these results suggest that the Spls do not modulate ScpA or Aur activity.

### Proteomic studies reveal differences between USA300 WT and Δ*spl*::*erm* mutant strain secreted and surface proteins.

Finally, the surface and secreted proteomes of the USA300 WT and Δ*spl*::*erm* mutant strains were analyzed. The objective of these experiments was to identify changes in protein levels that might explain the altered virulence phenotype of the Δ*spl*::*erm* mutant and suggest potential *S. aureus* proteins that are degraded by the Spls. The proteomic data were analyzed with the Scaffold program using Fisher’s exact test, and all hits with a *P* value of ≤0.010 were considered significant. The surface proteomics identified 63 proteins that were detected with increasing abundance in the Δ*spl*::*erm* mutant relative to USA300 WT (Table 1) and 21 proteins detected with decreasing abundance in the Δ*spl*::*erm* mutant (Table 2). A few genes involved in virulence and immune evasion were increased in the Δ*spl*::*erm* mutant, including those encoding IsdA and SpA. IsdA promotes *S. aureus* adherence to desquamated nasal epithelial cells (38), fibrinogen, and fibronectin (39) and enhances *S. aureus* survival of bactericidal fatty acids and peptides in human skin (40). SpA binds to IgG, which protects *S. aureus* from opsonophagocytosis and dysregulates the host adaptive immune response (41, 42). The cell wall-associated protein EbpS was also increased on the surface of *spl* mutant bacteria. Although EbpS was once thought to act as an adhesin by binding to elastin, recent studies have found that its contribution to bacterial adhesion is minimal (43). However, EbpS does mediate Zn^2+^-dependent growth and biofilm formation (44). Further, an investigation of the differential expression of *S. aureus* genes in murine models of colonization and pathogenesis found that *ebpS* was significantly upregulated in the blood relative to the nares (45). Therefore, this protein may play a role during infection. Increased levels of these proteins could enhance *S. aureus* immunomodulation and virulence.

**TABLE 1:** Proteins increased on *spl* mutant surface

GenBank (UniProt) accession no.	Name	Biological function(s)	Avg no. of assigned spectra		*P* value^a^
			WT	Δ*spl*::*erm*	
Q2FGW1 (EBPS_STAA3)	Elastin-binding protein EbpS	Adhesion, biofilm formation	24.00	47.67	0.00000021
Q2FHV1 (ISDA_STAA3)	Iron-regulated surface determinant protein A IsdA	Adhesion, immune evasion	113.33	136.00	0.0018
Q2FDT8 (ISAA_STAA3)	Transglycosylase IsaA	Autolysis	37.67	60.33	0.000013
Q2FGW9 (Q2FGW9_STAA3)	DNA-binding protein HU/Hup	Housekeeping, DNA structure	40.67	68.67	0.00000046
Q2FF94 (CH10_STAA3)	10-kDa chaperonin GroS	Housekeeping, protein folding	5.67	12.67	0.0022
Q2FDV8 (CLPL_STAA3)	ATP-dependent protease ClpL	Housekeeping, protein turnover	2.33	13.00	0.00000054
Q2FGB6 (GREA_STAA3)	Transcription elongation factor GreA	Housekeeping, transcription	115.33	153.67	0.0000033
Q2FJA3 (RL11_STAA3)	50S ribosomal protein R11 RplK	Housekeeping, translation	250.00	320.67	2.4E−09
Q2FFJ4 (GATC_STAA3)	Aspartyl/glutamyl-tRNA(Asn/Gln) amidotransferase subunit C GATC	Housekeeping, translation	41.67	57.00	0.0019
Q2FER1 (IF1_STAA3)	Translation initiation factor IF-1 InfA	Housekeeping, translation	15.33	26.33	0.0011
Q2FEQ4 (RL6_STAA3)	50S ribosomal protein L6 RplF	Housekeeping, translation	8.33	14.67	0.01
Q2FFJ6 (GATB_STAA3)	Aspartyl/glutamyl-tRNA(Asn/Gln) amidotransferase subunit B GatB	Housekeeping, translation	0.00	6.00	0.0002
Q2FF08 (RL31B_STAA3)	50S ribosomal protein L31 type B RpmE2	Housekeeping, translation	2.67	7.00	0.0096
Q2FEQ7 (RL30_STAA3)	50S ribosomal protein L30 RpmD	Housekeeping, translation	0.00	2.33	0.007
Q2FJU4 (Q2FJU4_STAA3)	Triacylglycerol lipase GehB/SAL2	Lipase	8.67	32.33	1.2E−11
Q2FH26 (ODO2_STAA3)	Dihydrolipoyllysine residue succinyltransferase component of 2-oxoglutarate dehydrogenase complex OdhB	Metabolism, amino acid degradation	0.00	2.33	0.007
Q2FHD0 (Q2FHD0_STAA3)	Glutamine synthetase GlnA	Metabolism, amino acid metabolism, glutamine synthesis	26.33	47.33	0.0000045
Q2FDV3 (Roca_STAA3)	1-Pyrroline-5-carboxylate dehydrogenase RocA	Metabolism, amino acid metabolism, proline catabolism	0.00	31.67	5E−30
Q2FJC8 (Q2FJC8_STAA3)	Cysteine synthase CysK	Metabolism, amino acid synthesis	82.33	133.00	7.4E−11
Q2FH38 (Q2FH38_STAA3)	Diaminopimelate decarboxylase LysA	Metabolism, amino acid synthesis	0.00	2.33	0.007
Q2FF22 (ATPA_STAA3)	ATP synthase subunit alpha AtpA	Metabolism, ATP synthesis (general metabolism)	22.67	32.00	0.01
Q2FKD2 (Q2FKD2_STAA3)	Acetoin (diacetyl) reductase SAUSA300_0129	Metabolism, carbohydrate metabolism	6.00	12.33	0.0052
Q2FK29 (LDH1_STAA3)	l-Lactate dehydrogenase 1 Ldh1	Metabolism, carbohydrate metabolism	0.00	6.00	0.0000028
Q2FFV5 (PCKA_STAA3)	Phosphoenolpyruvate carboxykinase PckA	Metabolism, carbohydrate metabolism, gluconeogenesis	12.67	35.00	0.000000003
Q2FG39 (Q2FG39_STAA3)	6-Phosphofructokinase PfkA	Metabolism, carbohydrate metabolism, glycolysis	18.00	36.67	0.0000028
Q2FDQ4 (ALF1_STAA3)	Fructose-bisphosphate aldolase class 1 Fda	Metabolism, carbohydrate metabolism, glycolysis	0.00	6.67	0.00000069
Q2FGM3 (Q2FGM3_STAA3)	6-Phosphogluconate dehydrogenase, decarboxylating Gnd	Metabolism, carbohydrate metabolism, pentose phosphate pathway	139.00	166.33	0.00072
Q2FFW0 (Q2FFW0_STAA3)	Transaldolase SAUSA300_1725	Metabolism, carbohydrate metabolism, pentose phosphate pathway	40.67	66.33	0.0000026
Q2FEZ2 (Q2FEZ2_STAA3) (+1)	Deoxyribose-phosphate aldolase DeoC	Metabolism, carbohydrate metabolism, pentose phosphate pathway	4.00	10.67	0.0013
Q2FHZ7 (Q2FHZ7_STAA3)	Phosphocarrier protein HPr/PtsH	Metabolism, carbohydrate metabolism, sugar PTS ^b^	22.67	50.67	1.7E−09
Q2FE56 (Q2FE56_STAA3)	NAD-dependent epimerase/dehydratase family protein SAUSA300_2387	Metabolism, cofactors and vitamins	7.67	14.33	0.0065
Q2FJM5 (GUAA_STAA3)	GMP synthase GuaA	Metabolism, nucleotide metabolism	0.00	8.00	0.00000004
Q2FI07 (PUR5_STAA3)	Phosphoribosylformylglycinamidine cyclo-ligase PurM	Metabolism, nucleotidemetabolism	0.00	2.67	0.0034
Q2FI11 (Q2FI11_STAA3)	Phosphoribosylformylglycinamidine synthase PurS	Metabolism, nucleotide metabolism	0.00	2.67	0.0034
Q2FI15 (FOLD_STAA3)	Bifunctional protein FolD	Metabolism, one-carbon metabolism, tetrahydrofolate interconversion	317.33	350.00	0.0017
Q2FFV8 (Q2FFV8_STAA3)	Oxidoreductase SAUSA300_1728	Oxidoreductase	99.67	123.33	0.00084
Q2FIB9 (Q2FIB9_STAA3)	NADH-dependent flavin oxidoreductase SAUSA300_0859	Oxidoreductase/FMN^c^ binding	0.00	3.00	0.0017
Q2FEB6 (Q2FEB6_STAA3)	Putative uncharacterized protein SAUSA300_2327	Putative FMN binding and pyridoxamine-phosphate oxidase (general metabolism/oxidation)	30.67	52.67	0.0000055
Q2FFZ4 (Q2FFZ4_STAA3)	Putative thioredoxin SAUSA300_1690	Putative housekeeping, redox homeostasis	246.67	285.67	0.00016
Q2FEY0 (Q2FEY0_STAA3)	Haloacid dehalogenase-like hydrolase SAUSA300_2102	Putative hydrolase activity	0.00	2.67	0.0034
Q2FFH4 (Y1902_STAA3)	Conserved hypothetical protein, putative lactonase SAUSA300_1902	Putative lactonase activity	9.33	22.33	0.00002
Q2FGJ2 (Q2FGJ2_STAA3)	Proline dipeptidase SAUSA300_1491	Putative peptidase activity (aminopeptidase)	3.67	10.00	0.0016
Q2FFL6 (Q2FFL6_STAA3)	Aminopeptidase PepS	Putative peptidase activity (aminopeptidase)	0.00	4.00	0.0002
Q2FG30 (Y1654_STAA3)	Uncharacterized peptidase SAUSA300_1654	Putative peptidase activity (dipeptidase)	1.00	19.67	3.4E−15
Q2FFM0 (Q2FFM0_STAA3)	Uncharacterized putative glycosyl hydrolase SAUSA300_1856	Putative peptidase activity (endopeptidase)	0.00	16.00	1.6E−15
Q2FI96 (Q2FI96_STAA3)	Conserved hypothetical protein SAUSA300_0882	Putative phospholipid-binding protein	0.00	2.33	0.007
Q2FIJ2 (OHRL_STAA3)	Organic hydroperoxide resistance protein-like protein SAUSA300_0786	Putative stress response, oxidative stress (putative)	0.00	2.33	0.007
Q2FJY6 (Q2FJY6_STAA3)	ESAT-6-like protein SAUSA300_0278	Secretion	22.33	33.00	0.0044
Q2FDH3 (DRP35_STAA3)	Lactonase Drp35	Stress response, cell envelope stress	735.67	768.33	0.0044
Q2FEV0 (ASP23_STAA3)	Alkaline shock protein 23 Asp23	Stress response, cell envelope stress/alkaline shock protein	6.00	25.67	1.3E−10
Q2FGH0 (SODM1_STAA3)	Superoxide dismutase (Mn/Fe) 1 SodA	Stress response, oxidative stress	2.00	14.00	0.000000028
Q2FHD4 (Q2FHD4_STAA3)	Glutathione peroxidase SAUSA300_1197	Stress response, oxidative stress	17.67	26.33	0.0089
Q2FIW1 (Q2FIW1_STAA3)	Conserved hypothetical protein SAUSA300_0664	Unknown	72.33	113.67	0.000000009
Q2FKC5 (Q2FKC5_STAA3)	Cell wall surface anchor family protein SAUSA300_0136	Unknown	51.67	77.33	0.000013
Q2FEJ7 (Q2FEJ7_STAA3)	Conserved hypothetical protein SAUSA300_2246	Unknown	50.33	75.00	0.000021
Q2FDZ7 (Q2FDZ7_STAA3)	Conserved hypothetical protein SAUSA300_2447	Unknown	53.00	73.67	0.00027
Q2FDH4 (Y2620_STAA3)	UPF0312 protein SAUSA300_2620	Unknown	7.67	26.33	4.4E−09
Q2FHB6 (Q2FHB6_STAA3)	Conserved hypothetical protein SAUSA300_1215	Unknown	4.00	19.67	3.1E−09
Q2FIT2 (Q2FIT2_STAA3)	Putative lipoprotein SAUSA300_0693	Unknown	18.00	33.00	0.000078
Q2FG19 (Q2FG19_STAA3)	GAF domain-containing protein SAUSA300_1665	Unknown	10.00	19.33	0.0011
Q2FKE8 (Q2FKE8_STAA3)	IgG-binding protein SpA	Virulence factor, immunomodulator	470.00	563.67	5.9E−10
Q2FHR3 (Q2FHR3_STAA3)	Antibacterial protein PSM-β2	Virulence factor, immunomodulator	31.00	42.33	0.0067
Q2FES8 (Q2FES8_STAA3)	MHC^d^ class II analog protein Map-w SAUSA300_2164	Virulence factor, immunomodulator	11.67	19.67	0.0056

a Fisher’s exact test.

b PTS, phosphotransferase system.

c FMN, flavin mononucleotide.

d MHC, major histocompatibility complex.

**TABLE 2:** Proteins decreased on *spl* mutant surface

GenBank (UniProt) accession no.	Name	Biological function(s)	Avg no. of assigned spectra		*P* value^a^
			WT	Δ*spl*::*erm*	
Q2FJ78 (SDRD_STAA3)	Serine-aspartate repeat-containing protein D SdrD	Adhesion, biofilm formation	2.33	0.00	0.0087
Q2FJ77 (SDRE_STAA3)	Serine-aspartate repeat-containing protein E SdrE	Adhesion, biofilm formation	34.33	22.67	0.0082
Q2FJ79 (SDRC_STAA3)	Serine-aspartate repeat-containing protein C SdrC	Adhesion, biofilm formation	317.67	32.67	0
Q2FHT6 (THIO_STAA3)	Thioredoxin TrxA	Housekeeping, cell redox homeostasis	7.67	0.67	0.000014
Q2FJA0 (RL7_STAA3)	50S ribosomal protein L7/L12 RpiL	Housekeeping, translation	81.00	45.67	0.00000015
Q2FKP7 (SYS_STAA3)	Seryl-tRNA synthetase SerS	Housekeeping, translation, aminoacyl tRNA biosynthesis	38.00	13.00	1.6E−09
Q2FH16 (Q2FH16_STAA3)	Glucose-specific IIA component crr	Metabolism, carbohydrate metabolism, sugar PTS^b^	163.67	108.33	0.000000038
Q2FJ55 (Q2FJ55_STAA3)	Phosphate acetyltransferase Pta	Metabolism, carbohydrate metabolism	14.67	6.67	0.0027
Q2FIU1 (Q2FIU1_STAA3)	Fructose 1-phosphate kinase FruB	Metabolism, carbohydrate metabolism	30.67	8.33	4.7E−10
Q2FIB3 (G6PI_STAA3)	Glucose-6-phosphate isomerase Pgi	Metabolism, carbohydrate metabolism	149.33	114.67	0.00061
Q2FEL8 (MOAC_STAA3)	Molybdenum cofactor biosynthesis protein MoaC	Metabolism, cofactors and vitamins	2.33	0.00	0.0087
Q2FJD1 (Q2FJD1_STAA3)	Hypoxanthine phosphoribosyltransferase Hpt	Metabolism, purine metabolism	538.00	36.00	0
Q2FHN7 (Q2FHN7_STAA3)	Dihydroorotase PyrC	Metabolism, pyrimidine metabolism	16.67	1.33	3.9E−11
Q2FHR1 (Q2FHR1_STAA3)	Uncharacterized N-acetyltransferase	Putative acetyltransferase	6.33	0.00	0
Q2FIC1 (PPI1_STAA3)	Putative peptidyl-prolyl *cis*-*trans* isomerase SAUSA300_0857	Putative housekeeping, protein folding	98.00	40.67	1.1E−16
Q2FG31 (Y1653_STAA3)	UPF0173 metal-dependent hydrolase SAUSA300_1653	Putative hydrolase activity	183.67	126.00	0.000000094
Q2FEC8 (Y2315_STAA3)	Conserved hypothetical protein SAUSA300_2315	Putative lipoprotein	16.67	5.67	0.000057
Q2FH36 (CSPA_STAA3)	Cold shock protein CspA	Regulation, transcription factor	12.33	5.00	0.0023
Q2FFR1 (TRAP_STAA3)	Signal transduction protein TRAP	Stress response, oxidative stress	143.33	100.67	0.0000086
Q2FEZ0 (Q2FEZ0_STAA3)	General stress protein 20U Dps	Stress response, starvation-inducible	74.67	53.00	0.0015
Q2FDL5 (Q2FDL5_STAA3)	*N*-Acetylmuramoyl-l-alanine amidase domain protein SAUSA300_2579	Unknown	81.67	47.33	0.00000044

a Fisher’s exact test.

b PTS, phosphotransferase system.

Proteins with decreased levels on the surface included the cell wall-anchored adhesins SdrC, SdrD, and SdrE. These proteins are members of the Clf-Sdr MSCRAMM family, and all have been demonstrated to promote *S. aureus* adhesion or immune evasion (46–48). SdrC self-associates to promote biofilm formation (49), while both SdrC and SdrD promote *S. aureus* adherence to desquamated nasal epithelial cells (38). SdrE inhibits classical (50) and alternative (51) complement activation. The decrease of these proteins in the *spl* mutant suggests that one or more Spl proteases indirectly affect their abundance, rather than directly degrading them.

Secreted proteomics identified 48 proteins increased (Table 3) and 28 proteins decreased (Table 4) in the *spl* mutant relative to the USA300 WT strain. As in the surface proteomics, IsdA was increased in the Δ*spl*::*erm* mutant secretome, suggesting that it is more abundant both on the cell surface and in shedding from the cell due to cleavage or cell lysis. Multiple virulence factors were also increased, including LukD, Sbi, Nuc, and Efb. LukD is part of the LukED bicomponent pore-forming toxin, which is cytolytic to several cell types, including monocytes and polymorphonuclear leukocytes (52, 53). LukED has also been reported to promote *S. aureus* growth in human blood by facilitating iron scavenging (54). Like SpA, Sbi binds IgG, which prevents opsonophagocytosis of *S. aureus* (55). Nuc is a potent secreted DNase that is able to degrade neutrophil extracellular traps (NETs), promoting *S. aureus* survival in the lung and host killing in a murine model of respiratory infection (56). NET degradation by Nuc also induces macrophage death (57). Efb is a secreted protein that binds fibrinogen and complement C3 to form a protective shield around *S. aureus* and inhibit its phagocytic clearance (58, 59). The increased production of these proteins in the Δ*spl*::*erm* mutant suggests that it may have enhanced immunomodulatory properties that contribute to its increased lethality in the rabbit model of pneumonia.

**TABLE 3:** Proteins increased in *spl* mutant spent medium

GenBank (UniProt) accession no.	Name	Biological function(s)	Avg no. of assigned spectra		*P* value^a^
			WT	Δ*spl*::*erm*	
Q2FJV7 (Q2FJV7_STAA3)	5′-Nucleotidase, lipoprotein e(P4) family SAUSA300_0307	Acid phosphatase	80.00	128.67	2.7E−09
Q2FHV1 (ISDA_STAA3)	Iron-regulated surface determinant protein A IsdA	Adhesion, immune evasion	7.67	23.67	0.00000036
Q2FF95 (CH60_STAA3)	60-kDa chaperonin GroL	Housekeeping, protein folding	12.67	32.67	0.00000014
Q2FF94 (CH10_STAA3)	10-kDa chaperonin GroS	Housekeeping, protein folding	0.00	2.33	0.0078
Q2FIM5 (CLPP_STAA3)	ATP-dependent Clp protease proteolytic subunit ClpP	Housekeeping, protein turnover	5.33	11.67	0.0055
Q2FJ98 (RPOB_STAA3)	DNA-directed RNA polymerase subunit beta RpoB	Housekeeping, transcription	0.00	2.67	0.0039
Q2FHI1 (EFTS_STAA3)	Elongation factor Ts/Tsf	Housekeeping, translation	80.33	122.00	0.00000021
Q2FES2 (RS9_STAA3)	30S ribosomal protein S9 RpsI	Housekeeping, translation	3.00	19.00	5.9E−10
Q2FG80 (RL21_STAA3)	50S ribosomal protein L21 RplU	Housekeeping, translation	5.67	14.67	0.00037
Q2FFJ4 (GATC_STAA3)	Aspartyl/glutamyl-tRNA(Asn/Gln) amidotransferase subunit C GATC	Housekeeping, translation	0.00	2.67	0.0039
Q2FEX8 (Q2FEX8_STAA3)	Glucosamine-fructose-6-phosphate aminotransferase (isomerizing) GlmS	Metabolism, amino acid metabolism	0.00	5.67	0.0000077
Q2FJ90 (Q2FJ90_STAA3)	Putative pyridoxal phosphate-dependent acyltransferase SAUSA300_0535	Metabolism, amino acid metabolism	0.00	2.33	0.0078
Q2FDQ7 (LDH2_STAA3)	l-Lactate dehydrogenase 2 Ldh2	Metabolism, carbohydrate metabolism, glycolysis	25.00	55.00	0.000000003
Q2FHY7 (Q2FHY7_STAA3)	Pyruvate dehydrogenase E1 component, alpha subunit PdhA	Metabolism, carbohydrate metabolism	12.33	26.67	0.000044
Q2FHY5 (Q2FHY5_STAA3)	Dihydrolipoamide acetyltransferase SAUSA300_0995	Metabolism, carbohydrate metabolism	5.33	14.33	0.00029
Q2FKD2 (Q2FKD2_STAA3)	Acetoin (diacetyl) reductase SAUSA300_0129	Metabolism, carbohydrate metabolism	0.00	2.33	0.0078
Q2FIB3 (G6PI_STAA3)	Glucose-6-phosphate isomerase Pgi	Metabolism, carbohydrate metabolism, glycolysis, gluconeogenesis	45.33	71.67	0.000014
Q2FIL9 (TPIS_STAA3)	Triosephosphate isomerase TpiA	Metabolism, carbohydrate metabolism, glycolysis, gluconeogenesis	27.00	52.00	0.00000063
Q2FE81 (GPMA_STAA3)	2,3-Bisphosphoglycerate-dependent phosphoglycerate mutase GmpA	Metabolism, carbohydrate metabolism, glycolysis, gluconeogenesis	13.33	33.67	0.00000014
Q2FGM3 (Q2FGM3_STAA3)	6-Phosphogluconate dehydrogenase, decarboxylating Gnd	Metabolism, carbohydrate metabolism, pentose phosphate pathway	29.33	44.33	0.0015
Q2FHZ7 (Q2FHZ7_STAA3)	Phosphocarrier protein HPr/PtsH	Metabolism, carbohydrate metabolism, sugar PTS^b^	41.00	105.50	1.4E−14
Q2FIQ8 (Q2FIQ8_STAA3)	Ribonucleoside-diphosphate reductase, beta subunit SAUSA300_0717	Metabolism, nucleotide metabolism	3.33	8.67	0.0057
Q2FI15 (FOLD_STAA3)	Bifunctional protein FolD	Metabolism, one-carbon metabolism, tetrahydrofolate interconversion	2.67	7.33	0.0081
Q2FJ56 (Y569_STAA3)	UPF0447 protein SAUSA300_0569	Putative heme-dependent oxidoreductase	0.00	9.00	7.5E−09
Q2FJP2 (Q2FJP2_STAA3)	Conserved hypothetical protein SAUSA300_0372	Putative lipoprotein	92.33	122.33	0.00022
Q2FKJ0 (Q2FKJ0_STAA3)	Putative lysophospholipase SAUSA300_0070	Putative lysophospholipase	0.00	5.00	0.000031
Q2FE21 (Y2422_STAA3)	Uncharacterized oxidoreductase SAUSA300_2422	Putative oxidoreductase	0.67	4.00	0.0065
Q2FEG8 (Q2FEG8_STAA3)	Oxidoreductase, short-chain dehydrogenase/reductase family GN=SAUSA300_2275	Putative oxidoreductase	0.00	3.00	0.002
Q2FFM0 (Q2FFM0_STAA3)	Uncharacterized putative glycosyl hydrolase SAUSA300_1856	Putative peptidase activity (endopeptidase)	11.33	22.67	0.0005
Q2FG28 (Y1656_STAA3)	Putative universal stress protein SAUSA300_1656	Putative stress response protein	3.33	12.33	0.00005
Q2FH36 (CSPA_STAA3)	Cold shock protein CspA	Regulation, transcription factor	5.33	27.33	3.7E−12
Q2FJY5 (Q2FJY5_STAA3)	Type VII secretion system protein EsaA SAUSA300_0279	Secretion	0.00	2.33	0.0078
Q2FEV0 (ASP23_STAA3)	Alkaline shock protein 23 Asp23	Stress response, cell envelope stress/alkaline shock protein	120.00	173.67	0.000000029
Q2FGH0 (SODM1_STAA3)	Superoxide dismutase [Mn/Fe] 1 SodA	Stress response, oxidative stress	17.33	35.00	0.000014
Q2FDV8 (CLPL_STAA3)	ATP-dependent Clp protease ClpL	Stress response, thermotolerance	19.33	42.33	0.00000021
Q2FJK5 (Q2FJK5_STAA3)	Conserved hypothetical protein SAUSA300_0409	Unknown	2.33	12.00	0.0000045
Q2FGB1 (Y1572_STAA3)	Conserved hypothetical protein UPF0473 protein SAUSA300_1572	Unknown	0.00	6.00	0.0000038
Q2FJN1 (Q2FJN1_STAA3)	Conserved hypothetical protein SAUSA300_0383	Unknown	0.00	4.00	0.00025
Q2FFJ2 (Q2FFJ2_STAA3)	CamS sex pheromone cAM373 SAUSA300_1884	Unknown	1.67	5.67	0.0085
Q2FKC5 (Q2FKC5_STAA3)	Cell wall surface anchor family protein SAUSA300_0136	Unknown	0.00	3.33	0.00098
Q2FG32 (Q2FG32_STAA3)	Universal stress protein family protein SAUSA300_1652	Unknown	0.00	3.33	0.00098
Q2FEV8 (Q2FEV8_STAA3)	Transporter gate domain protein SAUSA300_2133	Unknown	0.00	2.67	0.0039
Q2FIK2 (Q2FIK2_STAA3)	Thermonuclease Nuc	Virulence factor	323.00	369.67	0.0011
Q2FIH7 (Q2FIH7_STAA3)	Staphylococcal enterotoxin Q Seq/SelQ	Virulence factor	21.00	56.00	1.6E−12
Q2FFA2 (LUKL2_STAA3)	Uncharacterized leukocidin-like protein 2 SAUSA300_1975	Virulence factor, cytolysin	202.33	230.33	0.01
Q2FFR9 (Q2FFR9_STAA3)	Leukotoxin LukD	Virulence factor, cytolysin	2.00	2.33	0.00000021
Q2FE79 (SBI_STAA3)	Immunoglobulin-binding protein Sbi	Virulence factor, immunomodulator	54.67	90.00	0.0000002
Q2FHS5 (Q2FHS5_STAA3)	Fibrinogen-binding protein Efb	Virulence factor, immunomodulator	0.00	5.00	0.000031

a Fisher’s exact test.

b PTS, phosphotransferase system.

**TABLE 4:** Proteins decreased in *spl* mutant spent medium

GenBank (UniProt) accession no.	Name	Biological function	Avg no. of assigned spectra		*P* value^a^
			WT	Δ*spl*::*erm*	
Q2FE04 (Q2FE04_STAA3)	Fibronectin-binding protein B FnBPB	Adhesion	5.67	1.00	0.0013
Q2FE08 (Q2FE08_STAA3)	Putative cell wall surface anchor family protein surface protein G SAUSA300_2436	Adhesion, biofilm formation (nonfunctional in SAUSA300)	193.33	126.67	4E−11
Q2FI25 (Q2FI25_STAA3)	Autolysin Atl	Autolysis	657.67	605.33	0.0043
Q2FJH7 (SLE1_STAA3)	*N*-Acetylmuramoyl-l-alanine amidase Sle1	Cell wall turnover	72.67	54.33	0.0027
Q2FDT8 (ISAA_STAA3)	Transglycosylase IsaA	Cell wall turnover	140.00	99.33	0.0000026
Q2FEP2 (RL2_STAA3)	50S ribosomal protein L2 RplB	Housekeeping, translation	6.00	0.67	0.0002
Q2FJP8 (RS6_STAA3)	30S ribosomal protein s6 RpsF	Housekeeping, translation	11.67	1.00	0.000000033
Q2FE84 (Q2FE84_STAA3)	Amino acid ABC transporter, amino acid-binding protein SAUSA300_2359	Membrane transporter	33.33	22.00	0.005
Q2FIL7 (ENO_STAA3)	Enolase Eno	Metabolism, carbohydrate metabolism, glycolysis	354.67	297.00	0.000037
Q2FJM5 (GUAA_STAA3)	GMP synthase (glutamine-hydrolyzing) GuaA	Metabolism, nucleotide metabolism, purine metabolism	18.00	9.67	0.004
Q2FFI6 (Q2FFI6_STAA3)	Staphopain A ScpA	Protease, cysteine protease	102.33	69.00	0.0000053
Q2FFT4 (SPLF_STAA3)	Serine protease SplF	Protease, serine protease	3.67	0.00	0.00000024
Q2FFT0 (SPLB_STAA3)	Serine protease SplB	Protease, serine protease	9.67	0.00	3.3E−18
Q2FFT1 (SPLC_STAA3)	Serine protease SplC	Protease, serine protease	15.00	0.00	2.8E−14
Q2FFS8 (Q2FFS8_STAA3)	Conserved hypothetical protein SAUSA300_1759	Putative β-lactamase	68.33	53.33	0.01
Q2FJK6 (Q2FJK6_STAA3)	Conserved hypothetical protein SAUSA300_0408	Putative surface protein	58.00	43.33	0.0065
Q2FHI3 (Cody_STAA3)	GTP-sensing transcriptional pleiotropic repressor CodY	Regulation, transcription factor	5.67	1.33	0.0036
Q2FI16 (Q2FI16_STAA3)	Chitinase-related protein SAUSA300_0964	Unknown	7.67	2.33	0.0026
Q2FEJ0 (Q2FEJ0_STAA3)	Secretory antigen precursor SsaA	Unknown	18.67	8.33	0.00037
Q2FI95 (Q2FI95_STAA3)	Putative surface protein similar to Map-w SAUSA300_0883	Unknown	279.00	238.67	0.00098
Q2FE76 (Q2FE76_STAA3)	Gamma-hemolysin component B HlgB	Virulence factor, cytolysin	190.67	153.33	0.0002
Q2FFA3 (LUKL1_STAA3)	Uncharacterized leukocidin-like protein 1 SAUSA300_1974	Virulence factor, cytolysin	153.00	105.33	0.00000013
Q2FF89 (Q2FF89_STAA3)	Delta-hemolysin Hld	Virulence factor, cytolysin, immunomodulator	15.33	2.67	0.000000067
Q2FHR4 (Q2FHR4_STAA3)	Antibacterial protein PSM-β1	Virulence factor, immunomodulator	459.33	383.00	0.0000017
Q2FFF8 (SCIN_STAA3)	SCIN	Virulence factor, immunomodulator	326.67	250.33	0.000000013
Q2FES8 (Q2FES8_STAA3)	MHC^b^ class II analog protein Map-w SAUSA300_2164	Virulence factor, immunomodulator	578.67	500.67	0.000012
Q2FHR3 (Q2FHR3_STAA3)	Antibacterial protein PSM-β2	Virulence factor, immunomodulator	210.00	86.67	6E−37
Q2FIH8 (Q2FIH8_STAA3)	Staphylococcal enterotoxin K Sek/SelK	Virulence factor, toxin	32.00	19.33	0.0013

a Fisher’s exact test.

b MHC, major histocompatibility complex.

In contrast, multiple secreted virulence factors are decreased in abundance in the *spl* mutant, including the Υ hemolysin component HlgB, the staphylococcal complement inhibitor (SCIN), and the δ hemolysin Hld (Table 4). HlgB is the F subunit of the Υ hemolysin pore-forming toxins (60–62). Hlg toxins have been shown to contribute to virulence in a murine model of septic arthritis (63) and to induce eye injury when directly injected intraocularly into rabbits (64), as well as promote *S. aureus* virulence in rabbit endophthalmitis infection models (65–67). SCIN is a secreted protein that inhibits complement activation by binding to C3 convertases (68, 69). The Hld cytolysin is translated from RNAIII, the regulatory RNA that is the effector of the staphylococcal *agr* system (70). Decreased RNAIII would indicate lower *agr* activation in the *spl* mutant; however, our finding that Hla and ScpA activities are not decreased in the *spl* mutant suggests that this is not the case (Fig. 3B and C). Altered Hld levels therefore may be due to a posttranslational modification.

## DISCUSSION

The Spl proteases are an intriguing topic because, despite evidence that they contribute to *S. aureus*-host interaction, their specific functions have not been identified. Structural and activity analyses of SplA, SplB, and SplD have revealed that these enzymes are likely highly specific, given that they have cleavage preferences at subsites beyond the P1 residue of cleavage (26, 71–73). Further, the Spls are not produced as zymogens but, in some cases, appear to require the binding of a preferred substrate in order to take on an enzymatically active conformation (72), as has been demonstrated by characterization of SplB activity (71). The goals of this study were to investigate the function of the *spl* operon in virulence and to identify possible substrates of the Spl proteases.

The virulence phenotype of the *spl* mutant is complex, since the mutant-infected animals had a statistically insignificant change in lethality but more localized, less disseminated lung damage. We tested SplA cleavage of predicted substrate mucin 16 (26) and found that it induces the shedding of this protein from CalU-3 cells. SplA may therefore promote *S. aureus* invasion and spreading by removing mucin 16 from epithelial cells. Since mucin 16 is found on ocular, airway, and female reproductive tract epithelial cells (19), this function could facilitate infection at multiple body sites. This finding is also the first report of an Spl enzyme potentially cleaving a host target.

Proteomic studies of the secreted and surface proteins present on the USA300 WT and *spl* mutant strains revealed many proteins altered in abundance. The fact that intracellular and housekeeping genes were identified is one caveat of this experiment, suggesting that cell lysis may have occurred during sample preparation. However, none of the proteins identified contain the consensus motifs that have been identified for substrates of the Spls. These are W/Y-L-Y-S/T for SplA (26), W-E-L-Q for SplB (71), and R-Y/W-P/L-T/L/I/V-S for SplD (73). We therefore cannot predict any specific hits that are directly cleaved. However, it is possible that the Spls are able to cleave substrates with some deviation from these consensus motifs. The abundance of some proteins may also be indirectly affected by the Spls. Alternatively, SplC or SplE could be responsible for some effects, since only SplA, SplB, and SplD cleavage preferences have been published. Considering that the amino acid sequences of SplD and SplF are 94.6% identical (3), their activities are likely identical.

The proteomic results show virulence factors in both the increased and decreased protein groups, so it is difficult to predict specific virulence factors that are responsible for the virulence phenotype observed. A few of the proteomic hits also do not match our *in vitro* studies. For example, ScpA was found to be decreased in the *spl* mutant (Table 4) but unaltered when we tested its activity with a specific ScpA substrate (Fig. 3C). The decrease in Hld also could correspond to lower RNAIII, indicating lower *agr* activation, but this was also not observed in our Hla and protease studies (Fig. 3B and C). Hld translation could therefore be altered, or it may be degraded posttranslationally. Hld is a member of the phenol-soluble modulin (PSM) family of peptides, which are known to be protease labile (74, 75). Thus, the lower Hld levels in the *spl* mutant could be due to degradation by an unknown protease.

Our findings have some similarities to a previous study that tested the virulence and proteomics of a total protease knockout of *S. aureus* (74). In a murine model of sepsis, the protease null mutant had increased lethality in mice but reduced invasion of organs. Proteomics also identified an increase in the abundance of *agr*-regulated virulence factors such as Hla and other leukocidins. Although we did not observe an increase in Hla, some of our proteomic results mirrored those of the previously reported study. For example, the previous paper reported increased abundance of Sbi, LukE, Seq, Geh, Efb, IsdA, IsaA, Spa, and EbpS in the protease null mutant, all of which were increased in our *spl* mutant as well (Tables 2 and 4). This suggests that the Spl proteases contributed to the previously reported phenotypes and corroborates the model in which secreted proteases modulate virulence factor production.

A recent study found that the Spls are important mediators of the host allergic airway response to *S. aureus* (8). In the serum of five healthy *S. aureus* carriers, strong IgG4 antibody binding to the Spls was observed. Purified SplD induced a Th2-type response in a tissue explant model with tissue from human nasal polyps. In a mouse allergy model, inhalation of SplD also induced eosinophil infiltration and IgE antibodies to SplD (8). IgG4, IgE, and other Th2-mediated responses are involved in allergic airway disease (75). Interestingly, many allergens that induce allergic responses in the lungs are proteases, including proteases produced by *Aspergillus fumigatus* (76) and mites and cockroaches (77). These findings indicate that, in addition to SplD, the other Spls may cleave substrates in airway cells and contribute to allergic airway disease and other immune responses in the airway. Our finding that SplA induces mucin 16 shedding adds to this model, as mucin 16 removal could facilitate the ability of the other proteases to act on host cell targets.

The Spls are clearly highly specific proteases, and their natural targets have been difficult to identify. However, mounting evidence suggests that they are expressed *in vivo* and affect host immune responses. Future work will tease apart the roles of individual Spls in interaction with the host and unravel their host effects, hopefully by identification of their cleavage substrates in the host.

## MATERIALS AND METHODS

### Strains and plasmids.

LAC is a USA300 CA-MRSA strain (78), and the erythromycin-sensitive version called USA300 WT (79) was used in this study. The LAC Δ*spl*::*erm* mutant strain was generated by phage transduction from RN6390 Δ*spl*::*erm* (3) into LAC with φ80α. Strains were cultured on tryptic soy agar (TSA) plates or in tryptic soy broth (TSB) at 37°C, with shaking at 200 rpm for broth culture.

The SplA purification plasmid pSK236/*splA*6xHis was kindly provided by Suzan Rooijakkers of the University Medical Center Utrecht. This plasmid contains C-terminally His6-tagged *splA* that is under control of the *S. aureus* scn (SCIN) promoter. The plasmid was moved by electroporation into *S. aureus* Newman to maximize SplA production, since *scn* is positively regulated by the *sae* system (80) and *sae* expression is enhanced in the Newman strain (81).

### Rabbit pneumonia model.

The rabbit pneumonia model was prepared in accordance with reference 18. The USA300 WT and Δ*spl*::*erm* mutant strains were grown overnight in TSB, washed once in sterile TSB, and resuspended at 1 × 10^10^ CFU/ml. Adult Dutch belted rabbits (Bakkom) were anesthetized with ketamine (25 mg/kg) and xylazine (20 mg/kg) administered subcutaneously. A ventral midline incision was made through the skin and into the trachea; a catheter was then inserted and fed into the lungs. Approximately 2 × 10^9^ CFU of washed bacteria in 200 µl of TSB was delivered through the catheter to each rabbit. Inocula were plated to confirm the dose given. The actual dose was 2 × 10^9^ to 3 × 10^9^ CFU. The incisions were closed, and the rabbits were monitored for 6 days. Over the course of the experiment, the rabbits were treated for pain with buprenorphine (0.05 mg/kg twice daily) through intramuscular injection. Rabbits were euthanized by intravenous injection of 1 ml of a mixture of the sodium salts of phenytoin and pentobarbital (Beuthanasia-D) either on day 6 or if they were found during the course of the experiment to be unable to right themselves or exhibit escape behavior. At the time of death, rabbit lungs were photographed; tissues were fixed (10% neutral buffered formalin), processed, and embedded; and tissue sections were stained with H&E and histopathologically scored in accordance with standard principles (82).

### SplA purification.

*S. aureus* Newman expressing pSK236/*splA*6xHis was grown overnight in 5 ml of TSB plus 10 µg/ml chloramphenicol, and then the entire culture was added to 200 ml of TSB and grown overnight. The spent medium was harvested by centrifugation and filtration, and 121 g of ammonium sulfate was slowly dissolved in the spent medium to achieve approximately 85% saturation. The sample was centrifuged at 30,000 × *g* at 4°C for 30 min to collect the pellet, which was dissolved in 10 ml of PBS and dialyzed into 50 mM Na_2_HPO_4_ with 0.3 M NaCl at pH 8.0 and then into the same buffer with 10 mM imidazole for loading onto the His-Select nickel affinity gel (Sigma). Batch purification with the His-Select nickel affinity gel was performed according to the manufacturer’s protocol. The final protein was dialyzed into PBS and stored at −80°C.

### Mucin 16 slot blot assay.

CalU-3 cells were seeded at 500,000/well into 24-well plates and cultured with 1 ml/well minimum essential medium (Gibco) containing 10% heat-inactivated fetal bovine serum (Gibco) and 1× nonessential amino acids (Gibco) at 37°C in 5% CO_2_ for 2 days. For the SplA-induced shedding experiment, purified SplA was added to fresh medium without serum at 500 µl/well to avoid serum cross-reactivity with the mucin 16 antibody. For inhibition of SplA, 3,4-DCI was added at a final concentration of 10 µM. The cells were incubated with SplA at 37°C in 5% CO_2_ for 2 h, and the medium was removed for Western blotting. For the slot blot assay, samples were loaded onto polyvinylidene difluoride (PVDF) with a vacuum and a slot blot apparatus. The PVDF was first equilibrated in methanol for 15 s, in distilled H_2_O (dH_2_O) for 2 min, and in TBS plus 0.5% Tween 20 for 5 min. It was then placed into the slot blot apparatus on top of two pieces of filter paper wetted with TBS plus 0.5% Tween, and the vacuum was turned on low to drain excess buffer. A 100-µl volume of each sample was loaded into the slots, and then the vacuum was turned on until the liquid was absorbed. TBS plus 0.5% Tween 20 at 100 µl/well was then loaded to wash any unbound sample from the wells. The membrane was dipped into methanol and then dH_2_O and then blocked for 2 h in 5% BSA in TBS plus 0.5% Tween 20. A primary mouse anti-CA 125 antibody was diluted 1:200 in blocking buffer and incubated for 1 h, and a horseradish peroxidase-conjugated goat anti-mouse secondary antibody was diluted 1:5,000 in blocking buffer and incubated for 1 h. The membrane was washed with TBS plus 0.5% Tween 20 between steps. The membrane was incubated in chemiluminescent substrate and exposed to film.

### Blood survival assay.

Blood survival of the USA300 WT and Δ*spl*::*erm* mutant strains was measured as previously described (83). Strains were subcultured and grown to mid-log phase (optical density at 600 nm [OD_600_] of 0.8) and centrifuged at 8,000 × *g* for 5 min. Bacteria were washed twice in TSB and resuspended to 1 ml. A 100-µl volume was added to 1 ml of heparinized human blood, and the culture was placed on a tumbler at 37°C for 3 h. Serial dilutions were plated on TSA and counted.

### Hemolysis activity assay.

An Hla activity assay was performed as previously described (84). Briefly, overnight cultures of the USA300 WT and Δ*spl*::*erm* mutant strains were subcultured at 1:500 in TSB and grown for 24 h. Spent medium from these cultures was serially diluted 2-fold across a 96-well plate. Defibrinated rabbit blood (HemoStat Laboratories) was centrifuged to pellet rabbit erythrocytes, which were washed three times in 1.1× PBS and resuspended to a final concentration of 3%. A 30-µl volume of each spent medium dilution was mixed with 70 µl of the erythrocyte solution in the 96-well plate and then incubated statically at room temperature for 1 h, after which the OD_630_ was measured. The OD_630_ was plotted versus the percent spent medium on a four-parameter logistic curve with the Prism program, and the midpoint of the curve (the percentage of medium needed to lyse 50% of the erythrocytes) was used as an indicator of activity.

### ScpA activity assay.

Overnight cultures of the USA300 WT and Δ*spl*::*erm* mutant strains were diluted to an OD_600_ of 0.1 in 25 ml of TSB and grown for 24 h. At selected time points, spent medium was collected and incubated with a fluorescent resonance energy transfer (FRET) substrate that is based on the CXCR2 ScpA cleavage site (34). The FRET assay was performed in accordance with reference 85. The substrate was resuspended to a concentration of 50 µM in 20 mM Tris-HCl, pH 7.4. Spent medium was also buffered to 20 mM Tris-HCl, pH 7.4. The substrate (25 µl) was mixed with 175 µl of buffered spent medium, and fluorescence (excitation, 490 nm; emission, 520 nm) measurements were taken every 2 min for 30 min with a Tecan plate reader. The activity at each time point was expressed as the slope (fluorescence/time).

### Aur and hemolysis plate activity assays.

Overnight cultures of the USA300 WT and Δ*spl*::*erm* mutant strains were spotted in 5-µl amounts onto blood agar (5% [vol/vol] rabbit blood and 3% Bacto agar) or milk agar (5% nonfat dry milk and 3% Bacto agar) plates. The plates were incubated overnight at 37°C, and pictures of colonies with zones of clearing were taken.

### Proteomics.

Overnight cultures of the USA300 WT and Δ*spl*::*erm* mutant strains were grown in TSB and harvested for secreted and surface proteomic profiling. Secreted proteome profiling was carried out in accordance with reference 86, and surface proteomic analysis was carried out in accordance with reference 87.
